# Sinomenine derivative YL064: a novel STAT3 inhibitor with promising anti-myeloma activity

**DOI:** 10.1038/s41419-018-1147-z

**Published:** 2018-10-25

**Authors:** Yingying Wang, Linlin Wu, Haiyan Cai, Hu Lei, Chun-Min Ma, Li Yang, Hanzhang Xu, Qi Zhu, Zhujun Yao, Yingli Wu

**Affiliations:** 1 Hongqiao International Institute of Medicine, Shanghai Tongren Hospital/Faculty of Basic Medicine, Chemical Biology Division of Shanghai Universities E-Institutes, Key Laboratory of Cell Differentiation and Apoptosis of the Chinese Ministry of Education, Shanghai Jiao Tong University School of Medicine, 200025 Shanghai, China; 2 Department of Hematology, Shanghai 9th People’s Hospital, Shanghai Jiao Tong University School of Medicine, 639 Zhizaoju Road, 200011 Shanghai, China; 3 State Key Laboratory of Coordination Chemistry, Jiangsu Key Laboratory of Advanced Organic Materials, School of Chemistry and Chemical Engineering, Nanjing University, 163 Xianlin Avenue, 210023 Nanjing, Jiangsu China

**Fig. 1:**
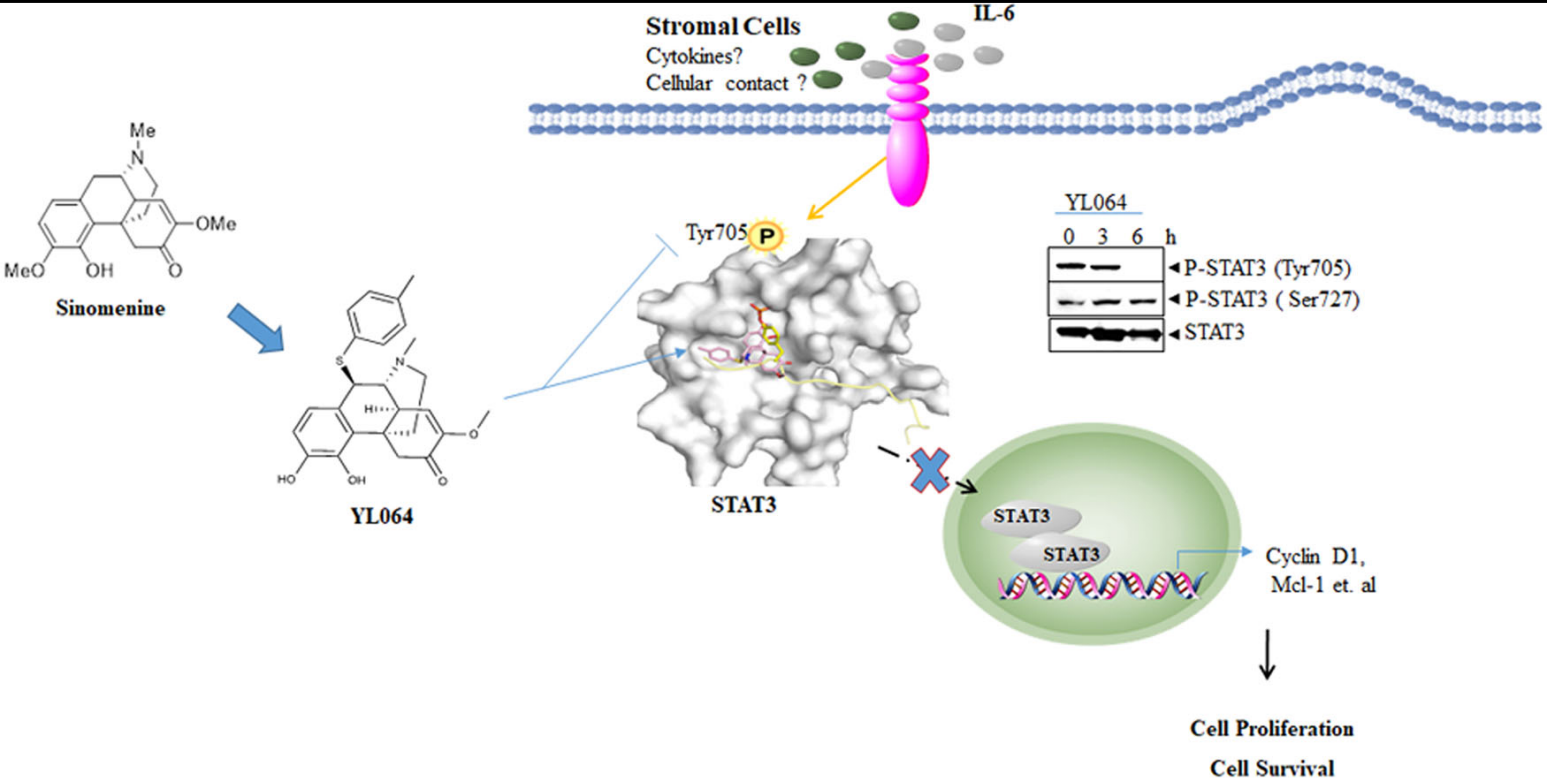
Model of the anti-MM effect of YL064. Different from other kinds of cancer cells, the survival and proliferation of MM cells is highly dependent on the bone marrow microenvironment. Bone marrow stromal cells can confer drug resistance to MM cells through direct contact and/or releasing soluble factors such as IL-6. Consequently, STAT3 is activated by tyrosine phosphorylation (Tyr705), followed by homodimerization, nuclear translocation, DNA binding, and subsequent expression of numerous gene products required for tumor cell survival (e.g., Mcl-1), proliferation (e.g., cyclin D1). YL064 is a derivative of sinomenine, a natural compound in clinical use. YL064 directly interacts with STAT3 through its SH2 domains, which in turn inhibits the Tyr705 phosphorylation of STAT3, and ultimately represses the proliferation and viability MM cells in vitro and in vivo. YL064 is a promising novel candidate STAT3 inhibitor for the treatment of MM

Multiple myeloma (MM) is the second most common form of blood cancer^1^. The introduction of proteasome inhibitors (e.g., velcade), immunomodulators (e.g., lenalidomide), and other novel agents have greatly improved the prognosis of patients with MM^2^. However, recurrence or drug resistance occurs frequently. In particular, the bone marrow microenvironment confers protection effect to MM cells by direct cell contact or releasing cytokines such as interleukin-6 (IL-6)^3^. Finding novel treatment is urgently needed. Signal transducer and activator of transcription 3 (STAT3) is a transcription factor that regulates the expression of many genes, such as Bcl-xL, Mcl-1, and Cyclin D1. STAT3 is involved in variety of biological processes, such as cell proliferation, differentiation, survival, inflammatory response, immunity, and angiogenesis^4^. In MM cells, STAT3 is aberrant activated by endogenous (e.g., IKK) or exogenous signals (e.g., stromal cells or IL-6) (Fig. 1)^5^. Targeting STAT3 is considered as a promising strategy against MM^6,7^.

Sinomenine is a natural compound isolated from Sinomeniumacutum and has been used clinically for the treatment of rheumatoid arthritis and other inflammatory diseases. However, in order to achieve efficacy, sinomenine must be used at high concentrations, which causes some side effects and limits its application^8,9^. In a recent article published in *Cell Death Discovery*^10^, we synthesized a series of sinomenine derivatives and identified YL064 as the most effective one. YL064 is at least 10-fold more potent than sinomenine in exerting cytotoxic effect against MM cells, but is not cytotoxic to normal blood cells even at higher concentrations. Importantly, YL064 can induce MM cell death in the presence of stromal cells or in the addition of exogenous IL-6. Furthermore, in vivo experiments shown that YL064 is well tolerated in mice and it could significantly inhibit tumor growth. These data suggest that YL064 is a promising lead compound for the treatment of MM. Due to the pivotal role of STAT3 in the pathogenesis of MM, we examined the possible effect of YL064 on STAT3 activation. Interestingly, YL064 can inhibit the phosphorylation of Tyr705 but not Ser727, indicating the suppression of STAT3 activity. In support of this, YL064 inhibited the nuclear translocation of STAT3 and the transcription of STAT3 target genes, including cyclin D1, Mcl-1. Intriguingly, both the endogenous activation and exogenous factors-induced activation of STAT3 could be blocked by YL064. Based on these data, we hypothesized that YL064 may directly interacts with STAT3. For this purpose, we synthesized Biotin-labelled or FITC-labelled YL064. The results showed that Biotin-YL064 could pull down STAT3 from cell lysate, which could be competed away by unlabeled YL064, indicating the specific interaction between STAT3 and YL064. And FITC-YL064 co-localized with STAT3 in MM cells. Moreover, using the cellular thermal shift assay^11,12^, we further demonstrated the engagement of YL064 with STAT3 in cells. Taken together, our data strongly suggest that YL064 directly interacts with STAT3 in cells. To reveal the mode of action of YL064, molecular docking studies were performed. The results showed that YL064 may prevent the interaction of STAT3 with phosphorylated tyrosine residues on cytoplasmic receptor kinases through targeting the SH2 domain of STAT3. However, a better understanding of the molecular basis for their interaction may be achieved by future co-crystal analysis.

In conclusion, using a chemical biological approach, we demonstrated that YL064 is a novel STAT3 inhibitor with potent anti-MM activity. Our data suggest that YL064 may overcome proteasome inhibitor resistance caused by the bone marrow microenvironment, which warrant further investigation.
